# Does renal denervation require cardiovascular outcome-driven data?

**DOI:** 10.1038/s41440-024-01598-7

**Published:** 2024-03-11

**Authors:** Syedah Aleena Haider, Max Wagener, Talha Iqbal, Shirjeel Shahzad, Paolo Alberto Del Sole, Niall Leahy, Darragh Murphy, Ruth Sharif, Ihsan Ullah, Faisal Sharif

**Affiliations:** 1 Department of Cardiology, University Hospital Galway, Galway, Ireland; 2 Department of Medicine, University of Galway, Galway, Ireland; 3 Department of Mathematics, University of Galway, Galway, Ireland

**Keywords:** Antihypertensive agents, Hypertension, Sympathectomy, Treatment outcome

## Abstract

Hypertension is a major driver of cardiovascular disease with a prevalence of 32–34% in adults worldwide. This poses a formidable unmet challenge for healthcare systems, highlighting the need for enhanced treatment strategies. Since 2017, eight major sham-controlled randomised controlled trials have examined the effectiveness and safety of renal denervation (RDN) as therapy for BP control. Although most trials demonstrated a reduction in systolic 24-hour/daytime ambulatory BP compared to control groups, open to discussion is whether major adverse cardiovascular events (MACE)-driven RDN trials are necessary or whether the proof of BP reduction as a surrogate for better cardiovascular outcomes is sufficient. We conducted an analysis of the statistical methods used in various trials to assess endpoint definitions and determine the necessity for MACE-driven outcome data. Such comprehensive analysis provides further evidence to confidently conclude that RDN significantly reduces blood pressure compared to sham controls. Importantly, this enables the interpolation of RDN trial endpoints with other studies that report on outcome data, such as pharmacological trials which demonstrate a significant reduction in MACE risk with a decrease in BP. Moreover, limitations associated with directly evaluating outcome data further support the use of BP as a surrogate endpoint. For example, conducting lengthier trials with larger numbers of participants to ensure robust statistical power presents a substantial challenge to evaluating outcome data. Thus, in light of the crucial need to tackle hypertension, there are notable advantages of considering BP as a surrogate for outcome data.

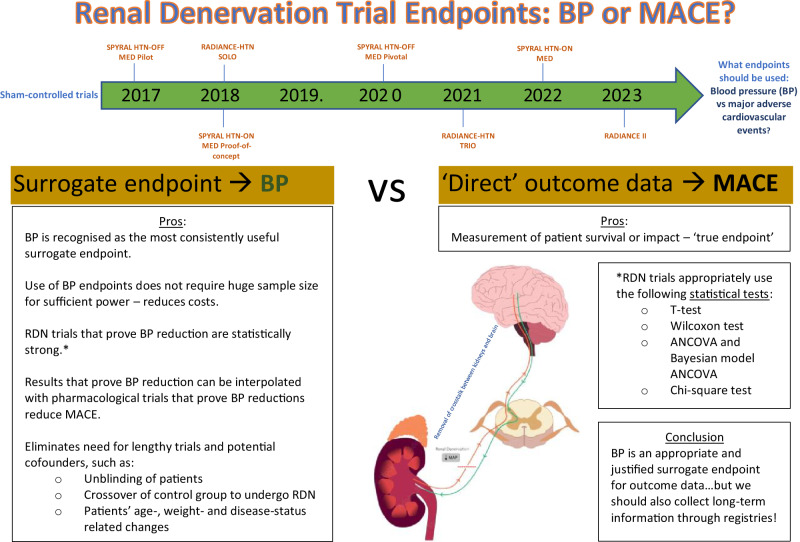

## Introduction

Hypertension (HTN) is the leading cause of cardiovascular morbidity and mortality worldwide, contributing to an estimated 8.5 million deaths in 2015 [1–3]. In the context of a rising prevalence and a high global age-standardised prevalence in adult women (34%) and men (32%), the World Health Organization aims to reduce the prevalence of HTN by 33% before 2030 [4–6].

However, there is an important mismatch between HTN awareness and its control, with only 18–23% achieving optimal control [4, 7]. Among a multitude of factors, poor patient compliance is a major contributory factor towards this unmet therapeutic target [8, 9]. In fact, a large-scale drug adherence study (*N* = 16,907) by Blaschke et al. showed a drop in medication adherence by up to 40% within the first year [10].

A particularly challenging group of patients are those suffering from resistant arterial HTN. These are patients under optimal medical treatment, with three or more antihypertensive drugs including one diuretic who fail to lower their office systolic and diastolic blood pressure (SBP and DBP) to <140 and/or <90 mmHg, respectively [11].

Device-based therapy for HTN offers an exciting and promising complementary intervention in the management of the condition, which may help diminish the well-documented issues associated with medication compliance [12]. Renal denervation (RDN) is one such example. It involves percutaneous ablation of the sympathetic renal nerves through the use of radiofrequency ablation, ultrasound or injection of neurotoxins such as alcohol. This interrupts the sympathetic cross-talk between the kidneys and the central nervous system. Specifically, blood pressure (BP) reduction occurs through reduced output from both afferent nerve fibres (leading to a reduction in sympathetic outflow to the heart, arterioles and kidneys themselves) and efferent nerve fibres (leading to a decrease in renin secretion, sodium absorption and vascular resistance) [13]. Crucially, such a procedure can potentially reduce BP throughout the 24-hour circadian cycle, described as an “always-on” effect independent of medication adherence and pharmacokinetics [14].

## History Of RDN

While the first ever in-human RDN procedure dates back to 1953, it was not until 2010 that the first generation of RDN randomised controlled trials (RCTs) was instigated [15]. Initial RCTs consisted of non-sham controls, with the implementation of sham controls in 2014 [16–19]. However, confounders such as frequent medication changes before and after randomisation in trials such as Symplicity HTN-3 limited the validity of results [19]. Furthermore, major methodological hindrances emerged primarily from limits in device design (unipolar vs. multipolar ablation catheters), varying medication burden, as well as the level of experience of interventionalists and the understanding of specific renal nerve lesion distributions [20–22].

Specifically, some renal nerves extend from sympathetic ganglia near the origins of the renal artery and travel on its surface, other renal nerves diverge away from the kidney artery before entering it, while the remainder join the renal arterial vessels after the first bifurcation of the main kidney artery. As a result, the ultrasound and neurolytic approaches are generally reserved to target the main renal artery, while radiofrequency ablation is used targets both the main artery and early branches.

Since 2017, refinements in subsequent second-generation trial designs culminated in a comprehensive report, published by the Hypertension Academic Research Consortium in 2022, focusing on clinical design principles and outcome definitions for studies evaluating device-based HTN therapies [23]. Owing to improved trial considerations and promising results, the European Society of Cardiology (ESC) Council on Hypertension in association with the European Association of Percutaneous Cardiovascular Interventions updated their guidance on the management of arterial HTN. In 2022, its clinical consensus document took a favourable stance towards RDN: *“This expert group proposes that RDN is an adjunct treatment option in uncontrolled resistant hypertension, confirmed by ambulatory BP measurements, despite best efforts at lifestyle and pharmacological interventions. RDN may also be used in patients who are unable to tolerate antihypertensive medications in the long term* [24].

Furthermore, the recent European Society of Hypertension guidelines also advise that RDN can be considered in patients with an eGFR >40 ml/min/1.73 m^2^ who have uncontrolled BP despite the use of antihypertensive drug combination therapy or if drug therapy elicits serious side effects and a poor quality of life, and in those with true resistant hypertension (class of recommendation II, level of evidence B) [25].

However, despite positive advancements, a lack of cardiovascular outcome-driven data represents a possible significant barrier to enabling the translation of RDN into clinical practice. Outcome-driven data refers to any information relating to patient survival or response in order to assess the effectiveness of an intervention [26]. Specifically in the case of RDN, cardiovascular outcome-driven data, henceforth referred to as ‘outcome data’, refers to changes in the rate of major adverse cardiovascular events (MACE) resulting from RDN-induced BP changes [27]. Examples of events include acute myocardial infarction, stroke and cardiovascular mortality which are widely used as study endpoints [28]. Indeed, without outcome data, there is scepticism around the clinical uptake of RDN and the question remains: do RDN-induced BP changes translate to meaningful clinical outcomes?

In this article, we will discuss whether there is a need for MACE outcome-driven RDN trials and whether BP reduction from RDN can be used as a surrogate for reductions in MACE. In order to do this, we will first review the robustness of the statistical methods that have been used to prove BP reduction in second-generation RDN trials and subsequently, we will consider the practicalities and limitations of potentially incorporating outcome data as an unequivocal indicator of treatment efficacy.

## Statistical analysis

Statistical analysis plays a key role in the medical domain, helping researchers and healthcare professionals to make informed decisions, draw meaningful conclusions, and improve patient care. Considering RDN studies, statistical analysis determines if there is any significant difference between the two study arms (RDN and sham procedure). Here we focus on the statistical techniques that have been used in the second-generation, sham-controlled RDN trials.

Table 1 outlines the eight latest published RCTs of the three currently available RDN devices (Radiance [ultrasound], Spyral [radiofrequency] and Peregrine [alcohol injection]) alongside their main corresponding statistical analyses. The unadjusted BP changes in the RDN and sham groups are reported, which are not always the primary outcome of each trial. Although six trials demonstrated a reduction in systolic 24-hour/daytime ambulatory BP compared to control groups, neither of the two latest trials (TARGET BP OFF-MED and SPYRAL HTN-ON MED Expansion) met their primary outcomes [29, 30]. The REQUIRE trial was not considered due to several shortcomings in the trial design (Including a lack of antihypertensive medication standardisation, medication adherence assessment and blinding) [31]. Additionally, note that the Spyral HTN-OFF MED Pivotal study included patients from the pilot study (SPYRAL HTN-OFF MED Pilot) [32, 33].

**Table 1:** Statistical summary of the second-generation, sham-controlled RDN trials

Trial	Year of publication	Number of centers	Sample size (male, female)	Randomisation ratio	Catheter device	Inclusion criteria	Primary efficacy endpoint	Question and corresponding statistical analysis		BP reduction: RDN vs control group
SPYRAL HTN-OFF MED Pilot [32]	2017	21	80 (57, 23)	RDN vs sham (1:1)	Symplicity Spyral - multi-electrode RF	Uncontrolled office and 24-hour BP in the absence of antihypertensive drugs	Change in 24-hour SBP at 3 months	Comparison of 24-hour and office SBP changes at 3 months, adjusted for baseline measures within sub-groups.	ANCOVA	−5.5 (95% CI: −9.1 to −2.0) vs −0.5 mmHg (95% CI: −3.9 to 2.90); *p* = 0.0414
								Between group differences and BP differences from baseline to the 3-month and 6-month follow-up time.	Unpaired and paired *t*-test	
								Comparisons between treatment groups were made to exact tests for multilevel categorical variables.	Chi-square test	
SPYRAL HTN-ON MED Proof-of-concept [35]	2018	25	80 (67, 13)	RDN vs sham (1:1)	Symplicity Spyral - multi-electrode RF	Uncontrolled office and 24-hour BP on 1 to 3 antihypertensive drugs	Change in 24-hour SBP at 6 months	Comparison of 6-month BP changes, adjusted for baseline measures within sub-groups.	ANCOVA	−9.0 (95% CI: −12.7 to −5.3) vs −1.6 mmHg (95% CI: −5.2 to 2.0); *p* = 0.006
								Between group differences and BP differences from baseline to the 3-month and 6-month follow-up time.	Unpaired and paired *t*-test	
SPYRAL HTN-OFF MED Pivotal [33]	2020	44	331 (220, 111)	RDN vs sham (1:1) with Bayesian adaptive design	Symplicity Spyral - multi-electrode RF	Uncontrolled office and 24-hour BP, in the absence of antihypertensive drugs	Change in 24-hour SBP at 3 months	Comparisons of BP changes between treatment groups, as well as pre-specified subgroup analyses, adjusted for baseline measures.	Bayesian ANCOVA	−4.7 (95% CI: −6.4 to −2.9) vs −0.6 mmHg (95% CI: −2.1 to 0.9); *p* = 0.0005
								Comparisons between treatment groups were made to exact tests for multilevel categorical variables.	Chi-square or Fisher’s test	
								Between group differences and within group BP differences from baseline.	Unpaired and paired *t*-test	
RADIANCE-HTN SOLO [60]	2018	39	146 (85, 61)	RDN vs sham (1:1)	Paradise - US	Uncontrolled daytime ambulatory BP in the absence of antihypertensive drugs	Change in daytime ambulatory SBP at 2 months	Treatment interactions: baseline vs daytime ambulatory SBP for subgroups (ethnicity, age, sex, geography, baseline daytime ambulatory SBP, baseline office BP, and abdominal obesity).	Linear regression model (ANCOVA)	−8.5 ± 9.3 vs −2.2 ± 10.0 mmHg; *p* = 0.0001
RADIANCE-HTN TRIO [36]	2021	53	136 (109, 27)	RDN vs sham (1:1)	Paradise - US	Uncontrolled office and daytime ambulatory BP on a three-drug fixed-dose combination pill	Change in daytime ambulatory SBP at 2 months	Exploratory analyses of pre-specified subgroups: change in daytime ambulatory SBP at 2 months as the dependent variable.	Linear regression analyses	−8.0 (IQR − 16.4, 0.0) vs −3.0 mmHg (IQR − 10.3, 1.8); *p* = 0.022
								Comparisons between treatment groups were made for continuous variables.	Unpaired t-test or Wilcoxon tests	
								Comparisons between treatment groups were made to exact tests for multilevel categorical variables.	Chi-square or Fisher’s test	
RADIANCE II [61]	2023	61	224 (160, 64)	RDN vs sham (1:1)	Paradise - US	Uncontrolled stage II hypertension (office and daytime ambulatory BP) in absence of antihypertensive drugs	Change in daytime ambulatory SBP at 2 months	Exploratory analyses of pre-specified subgroups: change in daytime ambulatory SBP at 2 months as the dependent variable.	Linear regression model (ANCOVA)	‒7.9 ± 11.6 vs ‒1.8 ± 9.5 mmHg; *p* < 0.001
TARGET BP OFF-MED [29]	2023	25	106 (78, 28)	RDN vs sham (1:1)	Peregrine system - ethanol injection via microneedles	Uncontrolled office and 24-hour BP, in the absence of antihypertensive drugs	Change in 24-hour SBP at 2 months	Comparison of office and 24-hour BP changes at 8 weeks, 3 months, 6 months and 12 months, adjusted for baseline measures within sub-groups.	ANCOVA	‒2.9 ± 7.4 (*p* = 0.009) vs ‒1.4 ± 8.6 mmHg (*p* = 0.25)
								Comparisons between treatment groups were made to exact tests for multilevel categorical variables.	Chi-square or Fisher’s test	
								Between group differences and BP differences from baseline to the 8-week, 3-month, 6-month and 12-month follow-up time.	Unpaired and paired *t*-test	
SPYRAL HTN-ON MED Expansion [30]	2023	56	337 (270, 67)	RDN vs sham (1:1 for initial 106 patients, 2:1 for subsequent 231 patients)	Symplicity Spyral - multi-electrode RF	Uncontrolled office and 24-hour BP on 1 to 3 antihypertensive drugs	Change in 24-hour SBP at 6 months	Comparisons of BP changes between treatment groups, as well as pre-specified subgroup analyses, adjusted for baseline measures.	Bayesian ANCOVA	−6.5 ± 10.7 vs −4.5 ± 10.3 mmHg; *p* = 0.12
								Comparisons between treatment groups were made to exact tests for multilevel categorical variables	Chi-square or Fisher’s test	
								Comparison of 6-month BP changes from baseline within groups	Paired *t*-test	

*BP* blood pressure, *RDN* renal denervation, *RF* radiofrequency, *US* ultrasound, *SBP* systolic blood pressure, *CI* confidence interval, *IQR* interquartile range

Each RCT is a patient- and outcome-assessor-blinded, sham-controlled, multicentre study that assesses ambulatory BP. Each of the statistical tests from Table 1 is used to assess whether the difference between patient groups is significant or non-significant. In order to scrutinise the validity of each trial’s statistical methods and subsequent BP reductions, it is essential to understand the principles underpinning each statistical technique. Each of the techniques is explained in the following subsections.

## T-test

The *t*-test is used to compare two independent samples containing continuous variables, assuming the data is parametric [34]. For example, in SPYRAL HTN-ON MED Proof-of-concept, it has been used to compare baseline BP between the RDN group and the sham control group [35]. On the other hand, a paired *t*-test is used to compare differences between two continuous dependent samples, assuming the data is parametric [34]. An example of its use is to compare baseline and 3-month BP changes between the same RDN intervention group, as is the case in SPYRAL HTN-OFF MED Pilot [32].

## Wilcoxon test

Using Wilcoxon tests in RDN research can also be valuable in assessing significant differences in continuous variables between groups, especially when dealing with data that does not adhere to normal distribution assumptions [34]. For example, in the RADIANCE TRIO (as well as subsequent analyses of RADIANCE SOLO), it has been used to compare antihypertensive dose and medication load between the RDN and control group, which is non-parametric data [36, 37].

## ANCOVA

Another statistical test is the analysis of covariance (ANCOVA), which is used in most trials to compare differences between three or more continuous variables, allowing control for confounding variables that can be continuous or discrete, assuming the data is parametric [38]. In fact, most included trials used ANCOVA to adjust for baseline blood pressure. In SPYRAL HTN-ON MED Proof-of-concept, ANCOVA has been used to compare the mean reduction in BP (continuous dependent variable) among patients who received different doses of medication (categorical predictor), while controlling for baseline BP (continuous covariate) [35]. Generally, ANCOVA is a combination of ANOVA and linear regression models, typically used to enhance the statistical power (i.e., the likelihood of detecting a significant difference between groups, if there is any) by reducing the variance in within-group errors. This can lead to more accurate estimates of group differences and a clearer understanding of the effects being studied [39]. The linear regression-based ANCOVA is a simpler and more deterministic approach that relies on traditional linear modelling assumptions (i.e., there is a linear relationship between the independent variables and the dependent variable and finds the best-fitting linear equation) [40].

However, SPYRAL HTN-OFF MED Pivotal 2020 have used a Bayesian model ANCOVA with an informative prior (updating previous findings with new data), allowing for the integration of data from both the pilot and pivotal trials in the primary analysis [33, 41]. This allows for a more sophisticated and informed analysis that leverages both previous knowledge and new data. This leads to more reliable and precise insights into the effectiveness and safety of the intervention, ultimately benefiting clinical decision-making [42]. The choice to select Bayesian-based ANCOVA analysis or linear regression-based ANCOVA analysis is dependent on the available data, and the underlying assumptions about the relationship between RDN and its outcomes.

## Chi-square test

The chi-square or χ2 test is used to compare differences between two or more samples containing discrete variables, assuming the data is non-parametric [34]. In Radiance-HTN TRIO, it has been used to make comparisons between treatment groups to exact tests for categorical variables such as sex and ethnicity [36]. Fisher’s Exact Test is a statistical test used to determine if there are nonrandom associations between two categorical variables, often employed when sample sizes are small and assumptions for larger tests (like chi-squared tests) might not hold [34].

Ultimately, the choice of statistical analysis depends on the research question, the nature of the data, and the specific goals of the RDN study. It is crucial to carefully assess the assumptions, limitations, and suitability of each method before deciding. For example, while utilising a *t*-test, researchers assume a normal distribution of the data and equal variances between groups, which makes the *t*-test sensitive to violations of these assumptions [42]. Further examples of ‘worst case’ scenarios in which these tests should not be used due to potential statistical errors are outlined in Table 2.

**Table 2:** Examples of worst-case scenarios for statistical analysis in renal denervation trials

Model	Examples of potential statistical errors
ANCOVA	• When the baseline blood pressure (covariate) shows no meaningful variation among the groups, rendering its inclusion unnecessary. • When the sample size is very small, making it difficult to meet the assumptions or obtain reliable estimates. • When the assumption of linearity between the covariates and the dependent variable is severely violated.
*T*-test	• When the data for the control and renal denervation groups have significant deviations from normality, and transformations do not resolve the issue. • When the sample size is extremely small, *t*-tests become less reliable and powerful with very few observations. • When there are multiple groups to compare, a *t*-test is only suitable for comparing two groups.
Fisher’s Model	• When there are very few categorical predictors or when the majority of cells have zero or minimal observations, leading to sparse data. • When the sample size is extremely small relative to the number of predictors, as overfitting may occur. • When the assumptions of the model, such as normality and homoscedasticity, are severely violated.

A comparison of the studies from Table 1 shows that the mean difference in BP is different for all the studies. One of the possible reasons for this is that different sample sizes and patient cohorts have been used. However, all the studies have concluded that BP reduction following RDN is significantly higher than the control (sham procedure). Without the actual data reported in the papers, we can assume that the authors have met and considered all the criteria and assumptions required while using these statistical techniques. Through these important statistical considerations, it is unlikely that the mistakes from Table 2 have been made. Thus, we agree with the findings that RDN significantly reduces BP when compared to sham controls.

## Device-based vs pharmacological BP therapy

Table 3 provides a summary of the potential advantages and disadvantages of using BP as a surrogate endpoint in RDN trials. Despite missing outcome data, the relative statistical strength of RDN trials may permit the use of BP changes as surrogates for cardiovascular endpoints. Weintraub et al. stated that the true endpoints in medicine are represented by health status, survival and cost, with any other measures simply serving as surrogate endpoints [43]. Therefore, a surrogate endpoint can be defined as an endpoint that predicts the occurrence and timing of a clinical endpoint of interest, in this case, MACE.

**Table 3:** Potential advantages and disadvantages of using blood pressure as an endpoint for outcome data in renal denervation trials

Advantages	Disadvantages
BP is historically recognised as one of the most consistently useful surrogate endpoints.	The ‘true endpoint’ (MACE) is not directly measured, with interpretation required for causality.
Smaller patient numbers are required for sufficient power which has positive financial implications.	Since RDN trials that use BP as a surrogate will have a shorter time span, they will be limited in their evaluation of safety endpoints.
Trials that demonstrate BP reduction via RDN have used statistically robust methods.	
Results that demonstrate BP reduction via RDN can be interpolated with pharmacological trials that prove MACE reduction through BP reduction.	
Trials with BP surrogate endpoints that do not show efficacy may obviate the need for endpoint trials, saving time, costs, and avoiding unnecessary patient risk.	
Trial durations are shorter which avoids potential confounders, including:	
▪ Unblinding of patients and outcome assessors, leading to performance bias	
▪ Crossover of control group to undergo RDN	
▪ Patients’ age-, weight- and disease-status related changes (i.e. longitudinal BP changes)	
▪ The addition of antihypertensives to medication regimes	
▪ Potential lifestyle modifications	

*BP* blood pressure, *RDN* renal denervation, *MACCE* major adverse cardiac events

Confidence that the use of a surrogate endpoint will result in an accurate inference requires prior rigorous validation of the surrogate. The statistical validity of a surrogate endpoint is a key consideration, first highlighted by Boissel et al. in their rigorous schema for surrogate endpoint evaluation [44]. That is, a surrogate is particularly useful if it is easily measurable and highly correlated with the true endpoint [45]. Fortunately, BP has often been recognised by multiple medical organisations as the most consistently useful surrogate endpoint [46, 47] Therefore, it may be possible to interpolate RDN trial endpoints with those from other studies that report on outcome data, such as pharmacological trials.

Of course, if RDN trials are to adhere to the same development and approval standards as other antihypertensive therapies, then it is necessary to acknowledge that many commonly recommended interventions lack outcome data. For example, the impact of BP reduction on cardiovascular outcomes through drugs such as alpha-1 adrenergic receptor antagonists and mineralocorticoid receptor antagonists has not been prospectively investigated [44]. This is also surprisingly the case for exercise- and metabolic surgery-based BP management. Nonetheless, BP reduction has been accepted as a surrogate for the reduction in MACE in all these interventions [44]. Further scepticism comes when considering other large, powered trials in which BP reductions from medications intended for the treatment of hypertension did not reduce MACE which raises the question of causality. For example, in the ALTITUDE trial, systolic and diastolic blood pressures were lower with aliskiren, a renin inhibitor, (between-group differences, 1.3 and 0.6 mm Hg, respectively) but there was no difference in cardiovascular endpoints [48].

However, a large-scale meta-analysis of 613, 815 patients by Etehad et al. has demonstrated that every medically achieved reduction of SBP by 10 mmHg is associated with a significantly reduced MACE risk (RR 0.80, 95% CI 0.77–0.83) [49]. Comparing patients with and without previous cardiovascular disease, a reduction in SBP by 5 mmHg is equally associated with significantly reduced hazard ratios for MACE (with cardiovascular disease HR 0.89, 95% CI 0.86–0.92; without cardiovascular disease HR 0.91, 95% CI 0.89–0.94) [48]. Assuming that the clinical benefit achieved through BP-lowering should not differ between device-based and medication-based BP reduction, pharmacological outcome data may have the potential to be used as a substitute for RDN outcome data. One could even argue that, when compared to pharmacological interventions, RDN may lead to fewer drug interactions and beneficial effects in other disease states involving sympathetic over-activity (including heart failure, atrial fibrillation, chronic kidney disease and metabolic syndrome). Of course, it is important to note that the exact mechanism of RDN is not understood, with a need to distinguish the cause and effects concerning the complex interplay between central and peripheral SNA, activation of the renin-angiotensin system, HTN and oxidative stress [50].

## Power and sample size

Perhaps a major obstacle to collecting outcome data for RDN trials is the sheer number of patients required. The ESC Council calculated that, in order for any antihypertensive trial to be sufficiently powered to evaluate outcome data, a minimum of 19,544 patients would be required. The calculation was for a power of 80% with a 2-sided alpha level of 0.05% and was based on an RCT that evaluated pharmacological interventions to reduce office systolic BP by 10 mmHg which conferred a 20% reduction in MACE [24, 51]. However, a relatively modest annual MACE baseline event rate of 3.5% was used in this calculation [52].

According to available literature, the use of a higher event rate, as is the case for high-risk patients, makes it easier to detect a statistically significant result which would render a smaller sample size requirement for sufficiently powered trials [53]. The inclusion of high-risk patients, in whom BP reduction confers the greatest absolute cardiovascular risk reduction, is a logical ‘next step’ for RDN trials [22]. It would be pertinent to calculate the new sample size requirements for RDN outcome data to re-assess feasibility.

Of course, it is critical to note that while an increased event rate reduces the required sample size, other factors such as the standard deviation of the dependent variable, the number of covariates, as well as the effect size, are required to calculate sample sizes [54]. These parameters are often estimated through assumptions and are not always clearly reported [54, 55]. The potential for attrition (loss of participants during the study) should also be considered but is often overlooked [56]. Such limitations should be considered since they can impact the accuracy of complex sample size calculations.

## Other considerations

Additionally, the evaluation of outcome data for RDN trials requires longer follow-up durations [25]. Aside from obvious cost implications, further difficulties pertain to the practicality of lengthier trials and the addition of confounding factors, as highlighted by the ESC [24]. For example, the eventual unblinding of patients, the addition of anti-hypertensive medications and the potential crossover of the control group to undergo RDN will influence outcomes. With time, patients’ age-, weight- and disease status-related BP changes may also obscure the true effect of RDN. This means that the use of BP as a surrogate for outcome data can therefore avoid these challenges.

However, whilst shorter trial durations are based on the premise that treatment-induced reductions in BP are associated with a long-term benefit on “hard” clinical outcomes, additional evidence is required to support this. A possible solution may be the demonstration of a regression in HTN-mediated target-organ damage in response to RDN. The creation of RDN registries which can report on long-term, follow-up data may be valuable in such circumstances, especially since the long-term safety of RDN warrants further investigation.

A major consideration in RDN trials pertains to the sustainability and time in therapeutic range (TTR) of BP reductions through RDN. TTR is the proportion of time that a patient spends within a specified, targeted BP range and has been recognised as an independent predictor of MACE amongst hypertensive patients [57, 58]. Again, the use of registries may be valuable as they can report on data such as TTR. The Global SYMPLICITY Registry is one such example, demonstrating encouraging results with sustained BP reductions and higher TTR through 36 months after RDN. Moreover, it has shown a significant correlation between TTR and MACE risk [59].

## Conclusion

The adoption of a standardized approach towards RDN trials through the inclusion of cardiovascular endpoints would undoubtedly facilitate the direct evaluation of RDN efficacy, especially since MACE ultimately constitutes the outcome of interest. This would permit the conduction of meta-analyses to achieve more powerful and comprehensive results that would further account for the safety profile of RDN.

However, in light of the higher costs and longer follow-up durations associated with the direct measurement of MACE endpoints, the implementation of well-designed registries that recruit larger patient cohorts may represent a potential compromise. Furthermore, although RDN trials do not directly contain outcome data such as MACE, their statistical robustness gives them the ability to interpolate results on BP reduction with pharmacological trials and avoid the significant impracticalities of including outcome data.

The overall aim of RDN trials is to reduce cardiovascular risk and improve patient outcomes. Therefore, outcome data from long-term clinical studies are crucial in determining the effectiveness of RDN in achieving these goals and in guiding clinicians to make informed decisions about the inclusion of RDN in their treatment strategies. Of course, considering the substantial global burden of HTN, the promising results of RDN trials and the barriers to direct analysis of outcome data, accepting BP as a surrogate for cardiovascular outcome data is likely to remain our primary option for gaining insight into the efficacy of RDN in the foreseeable future.
